# The mapping of active sites of amyloid‐targeting compounds through the utilization of immobilized fragments of amyloid hexamers

**DOI:** 10.1002/alz.088526

**Published:** 2025-01-09

**Authors:** Illhwan Cho, Soljee Yoon, Hye Yun Kim, YoungSoo Kim

**Affiliations:** ^1^ Yonsei University, Incheon Korea, Republic of (South); ^2^ Yonsei Institute of Pharmaceutical Sciences, Incheon Korea, Republic of (South); ^3^ Yonsei University, Incheon, Incheon Korea, Republic of (South); ^4^ Yonsei Institute of Pharmaceutical Sciences, Incheon, Incheon Korea, Republic of (South); ^5^ Integrated Science and Engineering Division & Department of Pharmac, Yonsei University, Incheon Korea, Republic of (South)

## Abstract

**Background:**

As amyloid‐β (Aβ) aggregates are considered as the biomarkers and key factors in the pathology of Alzheimer’s disease, there has been extensive investigation into Aβ‐targeting compounds for the development of diagnostics and drug discovery related to the disorder. However, the polymorphic and heterogenous nature of Aβ aggregates impedes the structural understanding of their structure. Consequently it is a major challenge to develop new diagnostic and therapeutic development of AD and to study the mechanism of Aβ‐targeting compounds.

**Method:**

Utilization of solid‐phase peptide synthesis, 37 Aβ hexameric Aβ fragments were synthesized. These fragments were immobilized on the 96‐well microplane to develop a mapping plate for investigating the active sites of Aβ‐targeting compounds. To confirm the mapping potential of our plate, previously reported fluorescent Aβ‐imaging agents (CRANAD‐28, bis‐ANS), Aβ aggregation inhibitors (curcumin, scyllo‐inositol), and Aβ aggregate dissociators (necrostatin‐1, sunitinib) were treated.

**Result:**

Using the mapping plate, we confirmed that CRANAD‐28 interacted with three domains of Aβ HHQKLVFF (Aβ_13‐20_), IIGLMV (Aβ_31‐36_), and GGVVIA (Aβ_37‐42_), and bis‐ANS were bound to the predominant parts of Aβ sequences except N‐terminal regions. We further determined that curcumin inhibited three distinct fragmental regions of Aβ (Aβ_14‐21_, Aβ_29‐35_, and Aβ_37‐42_) and scyllo‐inositol targeted VHHQKLVFF (Aβ_12‐20_), VGSNKGAIIGL (Aβ_24‐39_), and GGVVIA (Aβ_37‐42_) sites. Lastly, we confirmed that both necrostatin‐1 and sunitinib strongly dissociated GLMVGGVVIA (Aβ_33‐42_), the C‐terminal hydrophobic residue.

**Conclusion:**

This mapping plate can be employed to understand the active sites of Aβ‐targeting compounds, even in the lack of a comprehensive structural understanding of Aβ aggregates. Moreover, the insights gained from identifying targeting sites using this mapping plate can contribute to the rational approach in the development of diagnostic and therapeutic strategies for Alzheimer’s disease (AD).

